# Mortality reductions due to mammography screening: Contemporary population-based data

**DOI:** 10.1371/journal.pone.0188947

**Published:** 2017-12-20

**Authors:** James A. Hanley, Ailish Hannigan, Katie M. O’Brien

**Affiliations:** 1 Department of Epidemiology, Biostatistics, and Occupational Health, McGill University, Montréal, Québec, Canada; 2 Graduate Entry Medical School, University of Limerick, Limerick, Ireland; 3 National Cancer Registry Ireland, Cork, Ireland; University of Zurich, SWITZERLAND

## Abstract

Our objective was to compare breast cancer mortality in two regions of the Republic of Ireland that introduced a screening programme eight years apart, and to estimate the steady-state mortality deficits the programme will produce. We carried out age- and year-matched between-region comparison of breast cancer mortality rates, and of incidence rates of stage 2–4 breast cancer, in the eligible cohorts. The regions comprised counties that, beginning in early 2000 (region 1) and late 2007 (region 2), invited women aged 50–64 to biennial mammography screening. The data were supplied by the National Cancer Registry, Central Statistics Office. As impact measures, we used age-and-year-matched mortality (from breast cancers diagnosed from 2000 onwards), rate ratios and incidence rate ratios in the compared regions from 2000 to 2013. Ratios were adjusted for between-region differences in background rates. In cohorts too old to be invited, death rates in regions 1 and 2 were 702 per 0.91 and 727 per 0.90 million women-years respectively (Ratio 0.96). In the eligible cohorts, they were 1027 per 2.9 and 1095 per 2.67 (Ratio 0.88). Thus, rates in cohorts that could have benefitted were 9% lower in region 1 than region 2: (95%CI: -20%, +4%). The incidence rates of stage 2–4 breast cancer were 7% lower in region 2 than region 1 over the entire 14 year period, and 20% lower in 2007, i.e., before the screening in region 2 began to narrow the difference. Since mortality reductions due to screening only manifest after several years, the full impact of screening has not yet been realized in region 1. The lower rate observed in that region is a conservative estimate of the steady state benefit. Additional deaths would have been averted had screening continued beyond age 64.

## Introduction

Until recently, mortality reductions in randomized trials carried out 20–40 years ago were the only evidence for mammography screening. Since these trials, many countries have introduced mammography screening programmes, and ‘pre-post’ designs have been used to measure the change in mortality rates following these programmes. Some of the downward temporal trends may, however, have resulted from concomitant improvements in breast cancer management and treatment. A number of more complex designs, most commonly using a separate geographical area that did not introduce screening as a control, have been used to attempt to separate the effect of screening from the effect of improved treatment [1,2].

Using a ‘parallel’ design, a 2011 study [3] compared trends in mortality from breast cancer in three pairs of neighbouring European countries with similar population structures, socio-economic characteristics and access to treatment but where one country implemented a screening programme by 1990 with a later implementation in the matched country. It reported that Northern Ireland and the Republic of Ireland had similar reductions in age-standardized breast cancer mortality rates in the period 1989 to 2006 (29% vs. 26%) even though screening was introduced in Northern Ireland in 1990 and only in the eastern region of the Republic of Ireland in 2000. Unfortunately, the authors only had information on the age and year of death. Since they did not use incidence-files (available from cancer registries) that would have provided the year the cancer was diagnosed, their analysis included many deaths in women who could not have benefitted from the screening programme introduced to the first of the pair of countries: e.g., those who by the age/year it began, were either too old, or had already been diagnosed with breast cancer. Thus, by ignoring this first principle of cancer screening, their estimates of the benefit of the earlier introduction are greatly diluted.

What have come to be known as ‘incidence based’ mortality studies [4] avoid this bias by focusing on the mortality from breast cancer diagnosed *only after the first invitation to screening* and excluding deaths in women who could not benefit from the screening program i.e. whose cancers were diagnosed before screening was initiated. The term ‘incidence-based’ is a contraction of the more informative “incidence-file based mortality” term introduced by Chu et al. in 1994 [5]. The 2012 review of such studies, reported a breast cancer mortality reduction of 26% (95% confidence interval 13–36%) among women invited for screening and followed up for 6–11 years [6].

A country with a phased rollout of screening provides the opportunity for a geographical control group within the same country. Denmark and Norway are often referred to as ‘model countries’ for this type of comparison with the first phase of screening in Denmark offered to 20% of the target population in 1991–93 and a national rollout taking place between 2007 and 2010 [1,2]. Norway commenced screening in four counties in 1995 with a gradual expansion to the remaining 15 counties by 2005 [7,8].

The Republic of Ireland has a more recent history of a phased rollout of mammography screening with screening introduced in half of the country in 2000 (Phase 1, in “Region 1’) and the West and the South of the country only initiating screening at the end of 2007 (Phase 2, in ‘Region 2’). The program has two unusual features: screening is only until age 64, and the two regions involve similar sized populations. The primary objective of our study was to compare the contemporary breast cancer death rates in the two regions using data from the Irish National Cancer Registry on all breast cancer diagnoses since the first invitation to screening, in women aged 50–85, while correcting for any difference in the contemporaneous ‘background’ rates between the geographic regions involved in the two phases. Although differences in mortality are the most trustworthy metric, the delay between when screening is carried out and when mortality reductions become evident is long and variable. A second objective was therefore to use the incidence of stage 2–4 tumours, as a possible ‘leading indicator’ of the ultimate mortality reductions. Our third objective was methodological: we bring out the critical issue of ‘timing’ in any observed reductions in mortality or incidence of stage 2–4 tumours [9,10,11] by graphically representing the incidence rates and varying number of rounds of screening invitations received over time by different birth cohorts; the graphs indicates the relative magnitudes of, and the delays with which, the reductions are to be expected over time in the various birth cohorts. Throughout, we use graphical displays to remind readers of the principles underlying cancer screening.

## Methods

### BreastCheck–the Irish breast cancer screening programme

Following a pilot programme in the Dublin Region [12], the Republic of Ireland National Breast Screening Programme, BreastCheck, commenced in February 2000 for women aged 50 to 64 in the East of the country covering about 50% of the eligible population. The programme is by invitation, by district electoral division and county, to all women in this age range every two years and is free of charge. Eleven of the 26 counties commenced the programme in 2000, followed by a further three counties from 2004–2006. The remaining twelve counties in the West and South of the country commenced screening in December 2007. The target population acceptance rate of an invitation to screening has remained relatively stable over time ranging from 68% to 76% of those invited [13].

BreastCheck has offered a fully digitalised mammography service since 2008. Screening mammograms are taken in mobile digital screening units around the country or in dedicated units attached to local hospitals. Two view mammograms are taken and independently assessed by two consultant radiologists. Programme standards are based on European and national Guidelines for Quality Assurance in Mammography Screening[14,15] with radiologists required to read a minimum of 5,000 screening cases per year and have specific training in mammography. Women with abnormal results are invited for further investigation at an assessment clinic in the nearest screening unit. These investigations may include a further mammogram, ultrasound or biopsy. Women diagnosed with breast cancer are then referred to a multidisciplinary team.

### The Irish National Cancer Registry

The Irish National Cancer Registry began collecting data on all cancer diagnoses in the Republic of Ireland in 1994. Tumour registration officers are based in hospitals around the country and actively register new diagnoses. Most cases are identified through histopathology reports but the Registry also obtains records held in radiotherapy and oncology units, the hospital in-patient episodes (HIPE) database and death certificates. Death certificates are provided by the Central Statistics Office.

Completeness of case ascertainment by the Registry for breast cancer for women in the screening age group of 50–64 is estimated to be greater than 99%, through independent case ascertainment with BreastCheck records [16].

### Data and contrasts

We used data from the National Cancer Registry on all women aged 50–85, diagnosed with breast cancer from 2000 to 2013 and resident in the Republic of Ireland at the time of diagnosis. The diagnoses were linked with death certificates with follow up to the end of 2013 to ascertain date and cause of death.

Based on the county of residence at diagnosis, women were classified as having lived in Region 1 which consisted of the eleven counties (mostly in the East) which began screening in 2000 or Region 2 which consisted of the twelve counties which began at the end of 2007 (in the West and South). County of residence at diagnosis is assumed to reflect county of residence at invitation to screening. The 2011 census of the population reported 303,327 women aged 50 to 85 in counties comprising Region 1 and 282,736 women aged 50 to 85 in counties comprising Region 2. Quintiles of deprivation were derived from data in the 2002 census at electoral division level, and applied to individual women by linkage of address [17]. Within each quintile, we also compared the age-standardised breast cancer mortality rates for the five years, 1995–1999, before BreastCheck began.

All deaths attributed to breast cancers diagnosed since the year 2000, at ages of 50–84, in women in these two regions are included in the analysis, representing 94% of all breast cancer deaths in women aged 50–85 nationally in the time period of interest. The remaining 6% of deaths occurred in the three counties who introduced screening in 2004–2006.

The tumour registry only began collecting data in 1994, so we could not use ‘historical’ control groups such as those used in the studies from Denmark or Norway. Instead, we used ‘parallel’ control groups, and compared contemporary death rates in the two regions, but in women too old to benefit from a screening program limited to those aged 50–64.

### Statistical analysis

All graphics and calculations were custom-programmed in R. We used Lexis diagrams to display the basic data and to illustrate and report the results of the contrasts. To address our first aim, we carried out two (‘invitation-to-screen’) between-region contrasts: one rate ratio measured the amalgam of the effect of the earlier Region 1 screening and any inherent between-region differences in mortality rates even in the absence of screening; the other rate ratio measured just the regional difference in these ‘background’ mortality rates, caused by any regional differences in incidence or in quality of treatment. We then used the ‘double difference’ (a ratio of rate ratios) to estimate the ‘net’ difference attributable to the earlier introduction of screening in Region 1.

Thus, the first contrast involved breast cancer mortality rates in the *lower* portion of the Lexis diagrams, i.e. among women who had not been diagnosed with breast cancer by the age of 50, or by the year 2000, and were aged between 50 and 64 at some time between 2000 and the end of 2013. A summary Mantel-Haenszel rate ratio was computed using the 301 individual age-year cells as separate strata, and the variance of the log of this summary rate ratio was computed using the formula given in Breslow and Day [18]. Since the imbalances were minimal, the variance can be approximated as 1/D_1_ + 1/D_0_, where D_1_ and D_0_, are the overall numbers of deaths in the compared regions. The rate ratio for the second contrast, along with the variance of its log, was computed in the same way using the 203 cells in the *upper* portion of the Lexis diagrams, i.e. in the years lived by women aged 65 or older in 2000 who, likewise, had not been diagnosed with breast cancer by then, but were too old to benefit from a screening program that only extended to age 64. (Again, with ′ denoting the second contrast, the variance is well approximated by 1/D_1_′ + 1/D_0_′). Displayed ratios have been rounded to two decimal places. The variance of the log of the ratio of ratios, calculated as the sum of the two already-computed variances, was used to compute a confidence interval to accompany the point estimate of the ‘net’ reduction. This sum of variances is well approximated by the ‘Woolf-like’ formula (1/D_1_ + 1/D_0_ + 1/D_1_′ + 1/D_0_′), and shows that the width of the ultimate confidence interval is determined by the smallest of the 4 counts.

We also converted the (age-and-year-matched) double difference into an absolute number of deaths, so that the estimated number of invitations to avert one death from breast cancer could be determined.

To emphasize the time-patterns peculiar to cancer screening, and which ages in which years would be affected by screening, we plotted the location, in calendar time, age, and ‘years since last screening invitation’ of the numbers of deaths involved in each rate ratio in a separate figure. To bring out the patterns more clearly, we reduced the statistical noise by averaging the numbers of deaths in adjacent cells of the Lexis diagram.

Since only about 70% of invitees participated, the resulting ‘invitation to screen’ reduction is an average of the maximal effect in these 70% and the null reduction in the 30%. If one assumed that, had they participated, the 30% would have benefited to the same extent as the 70%, one can calculate that the reduction is approximately 100/70 = 1.4 times what is seen in the ‘invitation to screen’ analysis. We also addressed this ‘efficacy’ measure, since it is the relevant number for women who plan to participate fully.

To address our second and third objectives, we also used the between-region differences in incidence of stage 2–4 breast cancer as a ‘leading indicator’ of later mortality differences. The shorter lag between screening and the expected reductions in incidence—and the larger numbers of ‘events’—allowed us to examine the time patterns in more detail than was possible with mortality.

We analysed the patterns in two ways. First, we restricted the analysis to the 8 years 2000–2007, i.e., before screening began in Region 2. We grouped the Lexis cells both by attained-age and by birth-cohort, and used Poisson regression and Joinpoint regression [19] to compute *within-region* annual changes in each of seven ‘bands’, each 5 years wide. Second, we examined the *between-region* differences by computing 14 year-specific Mantel-Haenszel summary rate ratios, each one matched on attained age. In addition, in order to obtain smooth-in-time rate ratios, we used conditional Poisson regression. By fixing the total number of stage 2–4 cancers in an (age-at-diagnosis, year-of-diagnosis) Lexis cell, the number of them that arose from Region 1 can be treated as a binomial random variable [20]. The Region1: Region2 split within the cell is determined by the underlying ratio of the ‘background’ incidence rates in the two regions, the respective numbers of women-years they contribute, and the ‘net’ rate ratio–which itself can be modelled as constant-in or varying-in time.[19] The rate ratio for cell (a,y) has as its null value (unity) the rate of stage 2–4 tumours that, in the absence of a screening program, would have been diagnosed at stage 2–4 at age ‘a’ in year ‘y’. A ratio below unity indicates what fraction of these tumours would (*despite* the earlier invitations to screening) *still* have been diagnosed at stage 2–4 at age ‘a’ in year ‘y’ at this same age/year. One minus this rate ratio can thus be interpreted as the fraction of the initial ‘target’ that (as a result of the uptake of screening invitations) was detected in an earlier year—at stage 1 rather than at stage 2–4.

**Fig 1:**
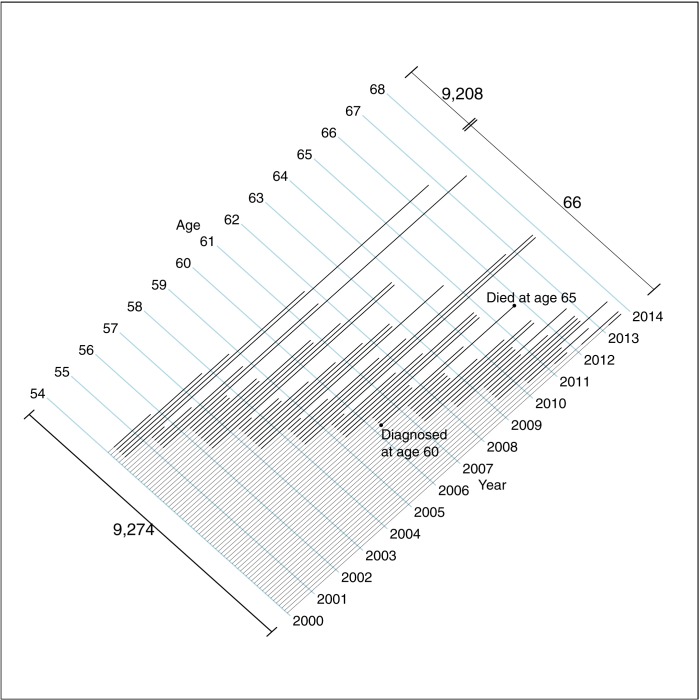
The ages when they were diagnosed with, and died of, breast cancer: 66 women in one selected cohort in region 2. Some 9,274 women, aged 54 in the year 2000, followed to the end of 2013. This cohort received just two screening invitations, at ages 62 and 64, too late to alter the course of these 66 fatal cancers. The lengths of the lighter portions of the lines are the maximal amounts by which screening might have advanced their diagnosis and treatment. Lines are drawn diagonally to orient readers to the full Lexis diagrams used in Figs 2 and 3.

By way of orientation to the results, Fig 1 shows, for one selected birth cohort in Region 2, the breast cancer deaths that a full screening program, i.e., *had it started in that region in 2000*, *might have averted*. Nine thousand women aged 54 residing in that region in 2000 only received their first (and last) invitation(s) to screening at age(s) 62 (and 64.) Over the 14 years from the beginning of 2000 until the end of 2013, a fatal breast cancer shortened the lives of some sixty six of these nine thousand women: some of these premature deaths, occurring before the women reached their late sixties, might have been averted had screening invitations commenced at age 54 (or 50) and moved the diagnosis and treatment to an earlier age/date.

## Results

**Fig 2:**
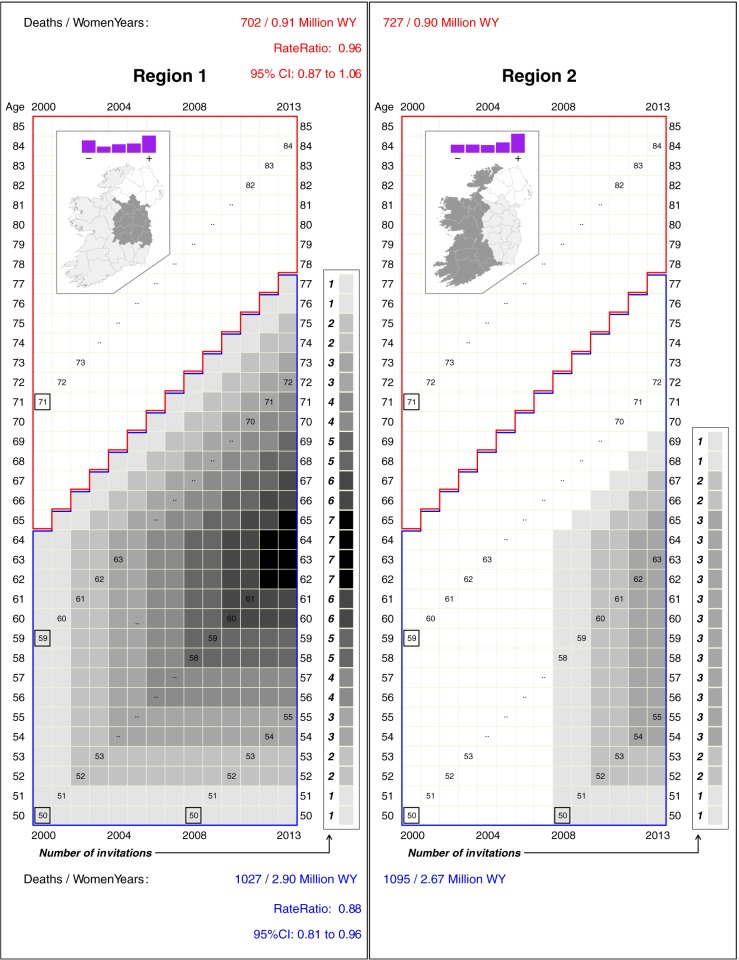
Numbers of screening invitations received by women in various birth-cohorts in regions 1 and 2, together with mortality rates and their ratios. Insets show the extent of each region, and (in purple) the fractions of those aged 50–85 in each quintile of the deprivation index, with ‘-‘ denoting the least and ‘+’ the most deprived. For each birth cohort, the numbers of screening invitations received by the end of the indicated years are indicated by squares ranging in colour from white (0) to black (7), and the numbers received by the end of 2013 are shown to the right of their last follow-up year. The *Region 1 vs*. *Region 2 comparison* limited to the years lived by women who were *65 or older in the year 2000* (cells inside the red boundary) measures the difference in the background mortality rates in the two Regions. The corresponding comparison limited to the years lived by women who were *64 or younger in the year 2000* (cells inside the blue boundary) measures the amalgam of this same (background) difference and the difference due to the greater duration of screening in Region 1.

The insets in Fig 2 show a summary of deprivation and the extent of each region. The higher proportion in the least deprived quintile in region 1 are mostly resident in or close to the largest city, Dublin. However, there was no consistent relation between quintiles of deprivation and rates of breast cancer mortality from 1995–99. Moreover, age-standardized mortality rates in the two regions in these 5 years pre-BreastCheck were quite similar to each other.

The main part of Fig 2 illustrates the numbers of screening invitations, ranging from none to seven, received by women in different birth-cohorts and Regions. In the age cohorts that were too old to be invited to screening [thus serving as a check on the comparability of the ‘background’ rates in two regions], the death rates in Region 1 and Region 2 were 702 per 0.91 million women-years (WY) and 727 per 0.91 million women-years, respectively (Mantel-Haenszel Rate Ratio 0.96). In the age cohorts that were eligible to be invited to screening, the corresponding death rates were 1,027 per 2.90 million women-years and 1,095 per 2.67 million women-years, respectively (Mantel-Haenszel Rate Ratio 0.88). Thus, adjusted for age, calendar year and the small between-region differences in background rates, the estimated mortality reduction *thus far* that might be attributed to the additional eight years of screening in Region 1 is approximately 9% (95% confidence interval: a 20% reduction to a 4% increase).

In absolute terms, the estimated number of deaths averted *thus far* by the additional eight years of screening (and 600 thousand more invitations to screening, if each invitation to one woman is counted as a separate invitation) in Region 1 is approximately 9 percent of 1095, or 100.

**Fig 3:**
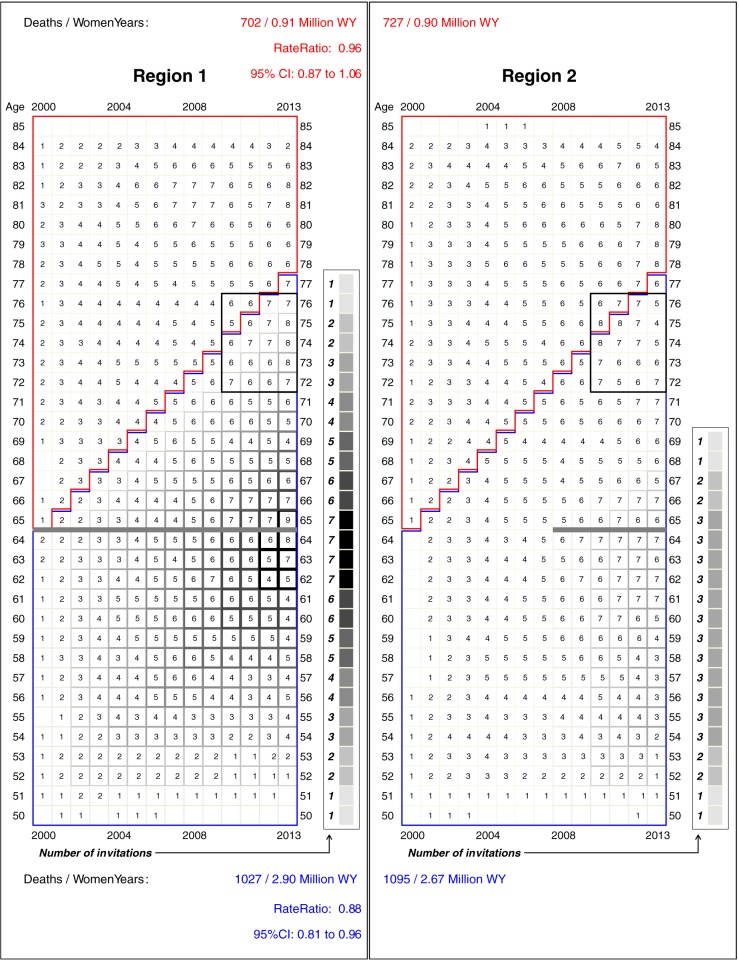
Numbers of deaths from breast cancer at each age and in each year *in region 1 and region 2*, plotted on the same grids as in Fig 1. The numbers in each cell have been smoothed, by averaging the actual numbers of deaths in the 9 cells in 3 x 3 square centered on the cell in question. To highlight the reach of screening that ends at age 64, also shown within the 4 x 5 rectangles, are the numbers of cancers that proved fatal at ages 72–76. For many of those in Region 1, the last screening invitation (shown by the thick grey horizontal line) was 8–12 years earlier.

To emphasize the complex time-patterns peculiar to cancer screening, and to highlight the age-window in which any additional mortality deficits generated by the planned extension of screening to age 69 would manifest, Fig 3 shows the location, in calendar time, age, and ‘years since last screening invitation’ of the numbers of deaths involved in each rate ratio. The small numbers of deaths in the early years reflect the ‘incidence-based’ nature of the comparisons; these occurred only a few years after diagnosis. The age locations of the later deaths in the as-yet-largely-unscreened counties in Region 2 provide a guide to the ages (several years earlier) at which screening-induced earlier treatment *might* have averted some of these deaths. Thus, for example, in the years 2010–2013 in Region 2, some 120 women died of breast cancer aged 72–76. None of their cohorts had been invited to screening. In the corresponding years and ages in region 1, some 116 died: the last invitation to their cohorts was when they were aged 64 i.e. 8 to 12 years earlier. The data in Fig 3 can also be used to determine the age at which the last screen would need to be to avert a portion of the still considerable numbers of deaths in women in their late 70s.

Table 1 shows the time-patterns in the incidence of stage 2–4 breast cancer over the first 8 years in each Region. In each of the 3 ‘horizontal’ and 4 ‘diagonal’ bands, the annual incidence decreased in Region 1 and increased in Region 2 (the results obtained by conventional Poisson regression and Joinpoint regression were identical)

**Table 1:** For each region, attained-age- and birth-cohort-specific annual changes in the incidence of stage 2–4 breast cancer in the 8-year period 2000–2007.

		Region 1	Region 2
Attained Age*			
50–54		-1%	+1%
55–59		-9%	+1%
60–64		-7%	+4%
Age in 2000** [**Born**]	**Followed to Ages**		
50–54 [1946–1950]	57–61	-3%	+5%
55–59 [1941–1945]	62–66	-7%	+5%
60–64 [1936–1940]	69–71	-3%	+4%
Reached age 50 in year**	**Followed to Ages**		
2001–2005 [1951–1955]	52–56	-5%	+3%

*The first three rows show results for 3 ‘horizontal’ age-bands, each five years wide: each band contains 12 of the total of 22 birth cohorts that traverse the 3 x 5 x 8 = 120 Lexis cells.

**The remaining rows group 145 Lexis cells by birth cohorts, into 4 ‘diagonal’ bands, each one 5 birth cohorts wide (the 3 cells traversed by the birth cohorts that reached age 50 in 2006 and 2007 are omitted).

Each ‘slope’, expressed as a percentage, was derived from the coefficient of ‘year’ in the fitted Poisson/Joinpoint regression.

**Fig 4:**
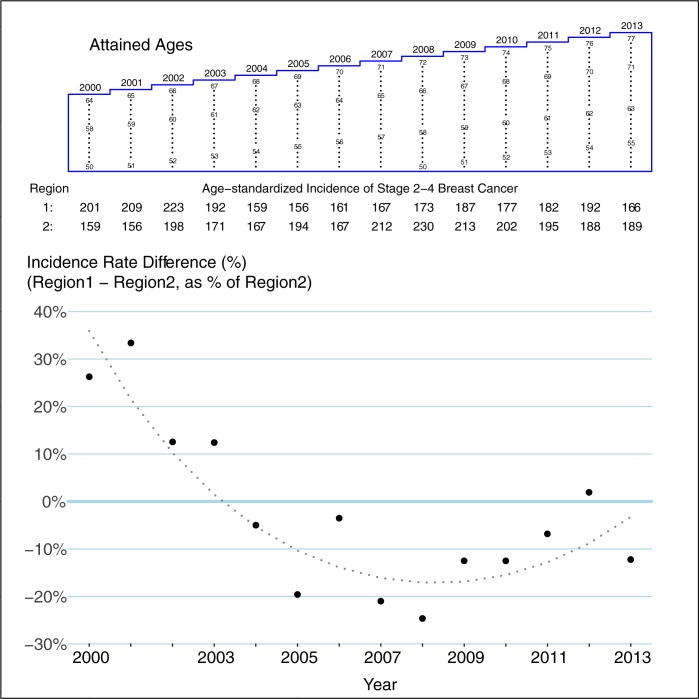
Year-specific differences between region 1 and region 2 in incidence rates (per 100,000WY) of stage 2–4 breast cancer. Yearly differences, expressed as percentages, are derived from age-matched Mantel-Haenszel summary rate ratios restricted to the cells within the blue polygon in the Lexis diagram (women who could have benefitted from screening). The smoothed differences are derived from a conditional Poisson regression (i.e. matched on age and year) that used all 36 x 14 = 504 Lexis cells, but that included a term to account for ‘background’ regional differences in the incidence (estimated from the cells enclosed in red in Fig 2), as well as linear and quadratic-in-time terms to allow the ‘net’ benefit of earlier access to screening in Region 1 to depend on year.

Over all 14 years in the 301 Lexis cells that could have been affected by screening, there were 5209 new cases of stage 2–4 breast cancer in the 2.90 million WY in Region 1, and 5117 in the 2.67 million WY in Region 2 (age-and year-matched Mantel-Haenszel rate ratio: 0.94). Adjusted for the (background) 1% higher Region-1 incidence in the 203 Lexis cells that could not have been affected by screening, the stage 2–4 incidence rate in those women who could have benefitted was thus 7% lower in Region 1 than Region 2. However, as is seen in Fig 4, that 7% ‘deficit’ is a not very meaningful average of the 14 year-specific impacts of the earlier screening in Region 1 than Region 2. The 20% Region1-Region2 difference in 2007 reflects the ‘deficit’ of stage 2–4 cancers in Region 1 in that year: the number that–in the absence of screening would have been diagnosed at stage 2–4 in 2007—had already been detected at an earlier stage in screening rounds 1–3 held in 2000–2005. This a 20% deficit is a good estimate of the long-term Region1-Region2 differences that would have been seen had Region 2 not begun screening at all. The narrowing of the differences in the final 5 years reflects the later Region 2 start: as time progresses, the 2 curves will converge.

Also included in the overall 7% difference is the *higher* rate of stage 2–4 cancers in Region 1 relative to Region 2 in the first 4 years. This also makes sense. Screening can detect stage 1 cancers that would otherwise have been diagnosed at stage 2–4 in a later year; but each round of screening (and especially the first few rounds) also detects stage 2–4 cancers that would otherwise have been diagnosed at stage 2–4 in a later year. A similar rise in the incidence of stage 2–4 cancers is evident in Region 2 when screening began there.

## Discussion

In order to weigh the benefits against the potential harmful effects such as over-diagnosis, those who fund organized mammography screening programs need to know how many future breast cancer deaths will be averted by them. The best estimates of benefit are to be found in the quasi-experimental data from contemporary population-based screening programs. Whereas all ‘big data’ need to be analysed carefully, cancer screening data, whether from the trials of decades ago or populations this millennium, need to be analysed even more carefully, to take account of the delayed returns on investment and other time-dependent features that are peculiar to cancer screening. Thus, we made extensive use of graphical displays and summarized the data using matching rather than modelling.

A synthesis in 2012 of the highest quality quasi-experimental studies–all based on incidence-based data—put the ‘best estimate’ of breast cancer mortality reduction produced by European service mammography screening programmes at 26% [6]. One contribution to this estimate were the D_1_ = 223 and D_1_ = 438 breast cancer deaths in the 10 years after/before screening was introduced to Copenhagen [1]. These two counts were the biggest component of the width of the confidence interval (D_1_ = 223, D_0_ = 2,333, D_1_′ = 438; D_0_′ = 2,123). Since the review, two additional data points have been added. The data from Norway, with 1,175 deaths in the 4 to 14 years after screening was introduced in the different counties, yielded a point estimate of 28% with the width of the CI determined by D_1_ = 1,175 and D_0_ = 8,996 [6]. The other data point was from the Funen area of Denmark with an estimate of 22% based on the 14 years after/before screening was introduced (D_1_ = 416; D_0_ = 566; D_1_′ = 4,246; D_0_′ = 4,111) [2].

The numbers of screening invitations issued to women in these areas followed the same pattern as on the left half of our Fig 2. No area had yet reached the steady state where every cohort had been invited since age 50, up until age 69, and followed until the full benefits of these screens–expected to be centred on the ages 55 to 75 or so–had been expressed. Thus the estimated reductions measure only *a portion* of what will be achieved in steady state, and each area provides a different portion: for example, in the Copenhagen [1] and Funen [2] studies, which relied on ‘time-shifts’ of 10 and 14 years respectively, the maximum number of invitations were only 5 and 7 respectively, and many cohorts had had far fewer.

What is different about these newest, Irish data, and how should the observed 9% difference between the regions involved in the two phases thus far be interpreted? The first difference is that the close to 50:50 sample size ratio in the two regions makes for a small variance (D_1_ = 1,027; D_0_ = 1,095 D_1_′ = 702; D_0_′ = 727). The second is that the correction in the *double* difference involves *contemporary* (post year 2000 only) rather than historical data. The closeness of the background rates in the women who were too old for the screening program reduces the risk of mixing mortality differences produced by screening and ones caused by regional differences in quality of care. Third, the Republic of Ireland is one of the few EU countries to have limited screening to women aged 50–64, rather than to those aged 50–69 as recommended by the Council of the European Union [21] (the program will now be extended to include all women from 50–69 by 2021).

The 9% regional difference seen thus far in Ireland measures how much of an advantage women in Region 1 have achieved thus far (i.e. in the 14 years) over their Region 2 counterparts (or their own counterfactuals) by *having had access to* organized screening *almost 8 years sooner*. The *full* effect in Region 1 (when all cohorts have received all 8 invitations, from age 50 to 64) could eventually be estimated indirectly if Region 2 had delayed its introduction, not for 8, but for say 20–25 years. Given our inexact knowledge as to the timing of the delayed cancer screening dividends, it is not possible to precisely extrapolate from the estimated 9% achieved this far with a lead of 8 years, to an estimate based on a (hypothetical) phase 1 lead of 20–25 years. However, one might extrapolate from the differences seen with the 10 and 14 year leads in the two Danish studies, as long as one allows for the shorter ‘age-reach’ of the Irish program (and its inability to avert most of the deaths that occur in women aged from the early 70s onwards). One should also allow for the initial phase 1 challenges in achieving full coverage and a 21–27 month cycle. Based on all of these considerations, it seems reasonable to project that *had the Region 1 lead been 20–25 years*, *their advantage over Region 2 in these years would have been close to 20*%–the remit of the program [13].

When magnified by a factor of 100/70 so that it refers to the benefit of full participation in region 1, the projection is closer to 30%.

The observed 9% difference that drives these estimates might have been attenuated by the phase 1 start up challenges, and by greater opportunistic screening in Region 2—prompted by awareness of the program in Region 1, and paid for by private health insurance. But this is offset by the possibly greater access to treatment in Region 1 pre 2008, when treatment pathways were linked to the screening programme. Centres of clinical excellence for cancer treatment were more common post 2008.

In addition to reporting the first 21^st^ century-only screening data thus far, this paper highlights an important but neglected principle in the analysis of cancer screening data. The effects of cancer screening are not like those of adult circumcision, where the resulting protection against HIV acquisition is immediate and lifelong, or one-time screening for abdominal aortic aneurysms [22], where the full benefits are already evident in year 2, and persist for at least a decade. To estimate the full effects of this activity/intervention, a difference of a year or two between starting phase 1 and 2 would have been more than adequate; to directly see the full mortality effects of eight rounds of every second year screening, a lead of perhaps 20–25 years is necessary.

The central role of timing and the difference in outlook between therapeutics and screening is further exemplified in the question of extending mammography screening from the ages of 50–64 to 50–69. When considering the implications of the last screening invitation being at age 69 rather than at age 64, it is more instructive to work *backwards*. Suppose that, in the absence of screening, a cancer had proved fatal at age 74; if there had been *just one* opportunity to screen for that cancer, at what age would it have been optimal to do so? What if there had been more than one? The patterns in Fig 2 and the raw data in Fig 3 illustrate why mortality data related to cancer screening, whether derived from old trials or newer quasi-experimental studies, need to be very carefully considered. Few analyses or meta-analyses to date have considered these core screening questions: how long after the beginning of screening do the mortality deficits manifest themselves? How long after the cessation of screening do the mortality deficits disappear? In each trial, how many rounds were there and how long was the follow-up? In light of these, what does a single average data from trials of varying screening duration and varying follow-up periods mean? And to whom does it apply?

The reductions produced by cancer screening cannot be summarized using a single number (the remit of the BreastCheck program was “reducing mortality from breast cancer by 20% in ten years”), but must be arrayed in time along both of the dimensions used in these Figs. Moreover, the cells must not be grouped merely by horizontal age-bands, as is commonly done. Women must also be *followed*, along the diagonals of the Lexis diagram, into *subsequent* age-bands. The good that screening at age 64 does only becomes apparent (as a mortality deficit) in *subsequent* age bands. These principles should be used to interpret not just these latest data from Ireland, but all of the trial and population data to date.

Reductions in the incidence of stage 2–4 tumours are not a prefect surrogate for the ultimate reduction in mortality. But the sharper and more immediate patterns are an important indicator, given that the gap between the two regions was only 8 years. Thus, the 20% ‘deficit’ in incidence of these tumours in Region 1, just before Region 2 began screening, is an encouraging finding.
